# Maternal mycotoxin exposure and adverse pregnancy outcomes: a systematic review

**DOI:** 10.1007/s12550-019-00384-6

**Published:** 2020-01-27

**Authors:** Nicholas N. A. Kyei, Daniel Boakye, Sabine Gabrysch

**Affiliations:** 1 Unit of Epidemiology and Biostatistics, Heidelberg Institute of Global Health, Heidelberg University, Im Neuenheimer Feld 324, 69120 Heidelberg, Germany; 2 Public Health Division, 37 Military Hospital, Accra, Ghana; 3 Division of Clinical Epidemiology and Aging Research, German Cancer Research Center (DKFZ), Im Neuenheimer Feld 581, 69120 Heidelberg, Germany; 4 Research Department 2, Potsdam Institute for Climate Impact Research, Potsdam, Germany; 5 Institute of Public Health, Charité – Universitätsmedizin Berlin, Berlin, Germany

**Keywords:** Pregnant women, Mycotoxins, Aflatoxins, Adverse pregnancy outcome

## Abstract

Mycotoxin exposure from food occurs globally but is more common in hot humid environments, especially in low-income settings, and might affect pregnancy outcomes. This study aimed to synthesize the evidence from epidemiological studies on the relationship between maternal or fetal exposure to different mycotoxins and the occurrence of adverse pregnancy outcomes. Multiple databases were systematically searched up to December 2018 to identify studies that assessed the association between mycotoxin exposure in pregnant women or fetuses and at least one pregnancy outcome. Studies were appraised and results were synthesized using standard methods for conducting systematic reviews. This review identified and included 17 relevant studies. There is some evidence to suggest that exposure to various *Aspergillus* mycotoxins (e.g., aflatoxin) during pregnancy may impair intrauterine fetal growth and promote neonatal jaundice. Findings were inconclusive concerning the influence of aflatoxin exposure on perinatal death and preterm birth. Only two studies assessed effects of maternal exposure to *Fusarium* mycotoxins (e.g., fumonisin) on adverse pregnancy outcomes. These studies found that maternal fumonisin exposure may be associated with hypertensive emergencies in pregnancy and with neural tube defects. Studies using grain farming and weather conditions as a proxy measure for mycotoxin exposure found that such exposure was associated with an increased risk of preterm birth and late-term miscarriage. In conclusion, there is already some evidence to suggest that exposure to mycotoxins during pregnancy may have detrimental effects on pregnancy outcomes. However, given the limited number of studies, especially on effects of *Fusarium* mycotoxins, more studies are needed for a more comprehensive understanding of the effects of different mycotoxins on maternal and fetal health and to guide public health policies and interventions.

## Introduction

The etiology of adverse pregnancy outcomes, such as miscarriage, preterm birth, low birth weight (LBW), and birth small-for-gestational-age (SGA), is multifactorial and not fully understood. Besides parity, maternal infection, hypertensive disease in pregnancy, and smoking as well-known risk factors (Kramer 2003), insufficient maternal nutrition has an undisputed impact on the outcome of pregnancies, as well as on the well-being of newborns (Kramer 2003; Ota et al. 2014). Furthermore, foods consumed by expectant mothers may contain hazardous natural products like mycotoxins. Mycotoxins are low-molecular-weight secondary metabolites of filamentous molds or micro-fungi that are toxic to vertebrates and other animals, even in low concentrations (Bennett and Klich 2003). Several hundreds of compounds are considered mycotoxins, but few are considered major threats to human and animal health, including aflatoxins (AFs), fumonisins (FBs), ochratoxin A (OTA), deoxynivalenol (DON), and zearalenone (ZEN) (Bennett and Klich 2003; Richard 2007; Smith et al. 2012). These mycotoxins are produced by a wide range of fungal species and often contaminate nuts, grains, and spices, especially in hot humid environments conducive to fungal growth (Chan-Hon-Tong et al. 2013). In general, *Aspergillus* mold strains produce AFs and OTA, while *Fusarium* mold strains produce FBs, DON, and ZEN (Bennett and Klich 2003; Smith et al. 2012).

Globally, mycotoxins contaminate an estimated 25% of cereal crops (CAST 2003) and due to their resistance to food processing and cooking practices, they are an almost ubiquitous exposure in cereal-consuming populations, especially in low-income countries (IARC 2015). In many of these settings with favorable environmental conditions for mold contamination, poor harvesting practices and improper storage of cereals and spices are common and food insecurity is widespread (FAO 2019). This is often coupled with less stringent food safety regulations and systems to protect consumers, leading to the consumption of highly contaminated foods with potentially serious health consequences (Bennett and Klich 2003).

Chronic exposure to AFs, the most widely studied mycotoxins, is a known risk factor for liver cancer (Turner et al. 2012) and has also been associated with low fertility in men (Shuaib et al. 2010a). Chronic exposure to FBs has been linked to esophageal cancer (Alizadeh et al. 2012; Chu and Li 1994; Wang et al. 2000), while OTA exposure is associated with kidney disease (Abid et al. 2003; Hope and Hope 2012; Raghubeer et al. 2017). Epidemiological studies from different regions have shown that mycotoxin exposure is widespread in pregnant women and newborns (Abdulrazzaq et al. 2002; Chan-Hon-Tong et al. 2013; Groopman et al. 2014; Jonsyn et al. 1995). This potentially harmful exposure during the critical first 1000 days of life of a child (Groopman et al. 2014) may be responsible for malnutrition and various adverse health outcomes (Etzel 2014). While maternal and infant exposure to AFs has, for instance, been shown to be associated with maternal anemia (Shuaib et al. 2010b), growth faltering (Gong et al. 2004; Turner et al. 2007), and severe acute malnutrition in children (McMillan et al. 2018), little is known about the effects of other mycotoxins and combinations of different mycotoxins on adverse pregnancy outcomes. This study therefore aimed to systematically review and synthesize the available evidence from epidemiological studies on the relationship between maternal mycotoxin exposure and adverse pregnancy outcomes.

## Materials and methods

### Literature search

A systematic literature search was conducted in six major databases including PubMed, Web of Science, Ovid-Medline(R), Ovid-EMBASE, Ovid-MIDIRS (Maternity and Infant Care), and Ovid-AMED (Allied and Complementary Medicine), without language restrictions, to identify relevant studies up to December 8, 2018. The review question was defined using the PECO (population, exposure, comparator, and outcome) framework (Morgan et al. 2018) as follows: “P: Among pregnant women or fetuses, what is the effect of; E: Detection of higher levels of different mycotoxins during pregnancy versus; C: No detection or lower levels of mycotoxins during pregnancy on; O: Specific adverse pregnancy and/ or newborn health outcomes.” For PubMed, the following search strategy was used, incorporating Text Words (TW) and Medical Search Headings (MeSH), and combining appropriate terms on pregnancy and pregnancy outcomes with terms on mycotoxins:

((“pregnant women” [tw] OR pregnancy [tw] OR pregnancy [mesh] OR mothers [tw] OR fetus* [tw] OR fetal [tw] OR fetal [mesh]) AND (“pregnancy outcome*” [tw] OR “pregnancy outcome” [mesh] OR miscarriage [tw] OR abortion [tw] OR prematurity [tw] OR “preterm birth” [tw] OR “preterm delivery”[tw] OR “low birthweight” [tw] OR “birth weight” [tw] OR “birth defect*”[tw] OR “fetal death” [tw] OR fetal death [mesh] OR “stillbirth” [tw])) AND (“fungal toxin*”[tw] OR aflatoxin*[tw] OR mycotoxin*[tw] OR mycotoxins [tw] OR mycotoxin*[mesh]).

For the search in Web of Science, instead of using text words and MeSH terms, a topic-specific search (TS) was conducted using the same search terms as above. For the Ovid database searches, a search was conducted using the same search terms as above combined with appropriate Boolean logic operators, incorporating the multiple field search (MP) and explosion (XM) function, as required. Additional searches for gray literature with the Google Scholar search engine, as well as cross-referencing, were conducted by manually searching bibliographies of the included articles for additional eligible studies. Finally, the search strategies for PubMed and Web of Science were saved and set up to receive automatic updates of new results. This systematic review was conducted in accordance with PRISMA guidelines (Liberati et al. 2009).

### Study selection

Studies were eligible for inclusion in this review if they (a) were published original observational studies or trials that assessed the effects of maternal (pregnant woman or fetal) exposure to mycotoxins (AFs, FBs, DON, OTA, ZEN etc.) on pregnancy outcomes (e.g. miscarriage, preterm birth, LBW and SGA) and (b) reported the association between maternal mycotoxin exposure and adverse pregnancy outcome(s) with an appropriate quantitative effect measure. Two reviewers (NNAK, DB) screened the titles and abstracts of identified articles to assess their eligibility for inclusion using the aforementioned criteria. Full texts of potentially relevant studies were obtained for further assessment. Studies that examined the prevalence of mycotoxins or of pregnancy outcomes only but not their association were excluded, as well as studies on the effects of mycotoxins more generally without a specific focus on maternal exposures and pregnancy outcomes. Relevant articles published as abstracts or posters only were also excluded because the amount of information was not sufficient for quality assessment.

### Methodological quality assessment and descriptive synthesis of results

Two investigators (NNAK, DB) extracted data from eligible studies independently from each other, using a pre-coded data extraction form. This included the basic characteristics of the included studies and information on methodological quality assessment, as reported in Table 1. A descriptive synthesis of included study results was then performed, as reported in Table 2. In case several different adjusted effect measures were presented in a study, the measures with the larger number of adjustment variables were extracted.

**Table 1:** Characteristics of studies on mycotoxins and adverse pregnancy outcomes

First author	Year	Country	Study design	Exposure assessment method	Sample size		Quality assessment*				
							RE	EA	OA	AD	Score
					Women	Infants					
De Vries	1989	Kenya	Prospective cohort	HPLC	184	92	0	1	1	0	2
Maxwell	1994	Nigeria	Cross-sectional	HPLC	–	625	0	1	1	0	2
Jonsyn	1995	Sierra Leone	Cross-sectional	HPLC	71	64	0	1	1	0	2
Sodeinde	1995	Nigeria	Case-control	HPLC	87	330	0	1	1	1	3
Ahmed	1995	Nigeria	Historical cohort + Case-control	HPLC	–	78^a^;124^b^	0	1	1	0	2
Kristensen	1997	Norway	Historical cohort	N/A-Census data	–	253,768	1	0	1	1	3
Abulu	1998	Nigeria	Cross-sectional	TLC	164	164	0	1	1	0	2
Kristensen	2000	Norway	Historical cohort	N/A-Census data	56,720	52,062	1	0	1	1	3
Moodley	2001	South Africa	Case-control	HPLC	51	–	0	1	1	0	2
Abdulrazzaq	2002	UAE	Cross-sectional	HPLC	201	201	0	1	1	0	2
Abdulrazzaq	2004	UAE	Historical cohort	HPLC	166	166	1	1	1	0	3
Missmer	2006	USA	Case-control	HPLC	409	–	1	1	1	1	4
Nordby	2006	Norway	Historical cohort	N/A-Census data	4912	–	1	0	1	1	3
Turner	2007	Gambia	Prospective cohort	ELISA	138	138	0	1	1	1	3
Shuaib	2010	Ghana	Cross-sectional	HPLC	785	–	0	1	1	1	3
Carlos	2014	Mexico	Case-control	N/A-dietary information	513	–	0	0	1	0	1
Lauer	2018	Uganda	Prospective cohort	HPLC	236	232	1	1	1	1	4

*UAE* United Arab Emirates, *USA* United States of America, *N/A* not applicable, *HPLC* high-performance liquid chromatography, *TLC* thin layer chromatography, *ELISA* enzyme-linked immunosorbent assay

^a^Sample size for cohort

^b^Sample size for case-control

*RE = Recruitment of study participants (0 = convenience/ non-random; 1 = random sampling method); EA = Exposure assessment (0 = subjective; 1 = objective); OA = Outcome assessment (0 = subjective; 1 = objective); AD = Adjustment for relevant factors (0 = no adjustment; 1 = at least one key potential confounding variable adjusted)

**Table 2:** Summary of findings on maternal mycotoxin exposure and adverse pregnancy outcomes

Author	Year	*N*	Mycotoxin(s)	Exposure assessment time	Source of specimen	Pregnancy outcome(s)	Results
De Vries	1989	208	Aflatoxins: • AFB_1_ • AFM_1_ • AFM_2_	- Prenatal - Perinatal	- Maternal blood - Cord blood	- Birth weight	• AF was detected in 53% of maternal blood samples in concentrations from 12 to 11,574 pg/ml, and in 37% of cord blood samples in concentrations from 17 to 6819 pg/ml. • Mean birth weight of female infants of AF-positive mothers was 255 g lower than that of females born to AF-negative mothers. • Mean birth weight of male infants of AF-positive mothers was 132 g higher than that of males born to AF-negative mothers. • There was an interaction between sex and AF in maternal blood at delivery on birth weight (ANOVA *p* = 0.020).
Jonsyn	1995	64	Aflatoxins: • AFB_1_ • AFM_1_ M_2_ • AFG_1_ G_2_ • Aflatoxicol Ochratoxin A	- Postnatal - Perinatal	- Cord blood - Maternal blood	- Birth weight - LBW	• AF was detected in 91% and OTA in 25% of cord blood in concentrations from 4 to 9000 pg/ml (AF) and 200 to 3500 pg/ml (OTA). • AFM_1_ (56%), aflatoxicol (53%), and AFG_2_ (41%) were the most frequently detected aflatoxins. • AF was also detected in 6 out of the 8 maternal blood samples analyzed. • LBW occurred in 17.2% of deliveries and was associated with higher prevalence of AF, and with significantly higher levels of AFM_2_ (*p* < 0.05) and AFG_2_ (*p* < 0.04). • AF levels in normal birth weight babies were higher in girls than in boys. • OTA levels were higher in LBW babies compared to normal weight babies (0.5 vs 0.9 ng/ml; *p* = 0.06). • Exposure to AF or OTA had no effect on the birth weight of boys. Mean birth weight of exposed girls was 190 g lower than of unexposed girls.
Sodeinde	1995	407	Aflatoxins: • AFB_1_ • AFM_1_ M_2_ • AFG_1_ G_2_ • Aflatoxicol	- Postnatal	- Infant blood - Maternal blood	- Neonatal jaundice	• AF was detected in 27.4% of jaundiced babies and 17.0% of their mothers, and in 16.6% of control babies and 14.4% of their mothers. • AFB_1_ was the most frequently detected aflatoxin and aflatoxicol was only detected in jaundiced babies. • AF was associated with higher prevalence of neonatal jaundice (adj OR 2.68; 95% CI 1.18–6.10).
Ahmed	1995	202	Aflatoxins: • AFB_1_ • AFM_1_ M_2_ • AF G_1_ G_2_ • Aflatoxicol	- Postnatal	- Cord blood - Infant peripheral blood	- Neonatal jaundice	• AF was detected in 30% of cord blood overall. • AF was detected in 38% of cord blood of babies who developed jaundice in concentrations from 13 to 238,177 pg/ml and in 23% of babies who did not develop jaundice in concentrations from 32 to 2654 pg/ml (*p* = 0.22). • Later in neonatal life, AF was detected in 24.3% of peripheral blood of babies who developed jaundice in concentrations from 24 to 23,749 pg/ml and in 30% of babies who did not develop jaundice in concentration from 51 to 3151 pg/ml (*p* = 0.76). • Mean AF concentrations were not higher in jaundiced than in non-jaundiced neonates (*p* = 0.87).
Abulu	1998	164	Aflatoxins: • AFB_1_ B_2_ • AFG_1_	- Postnatal	-Cord blood	- Neonatal jaundice - Birth weight	• AF was detected in 70% of cord blood samples. • AFB_1_ and B_2_ were the most frequently detected aflatoxins. • AF was detected in 80% of cord blood of jaundiced neonates and in 36% of cord blood of non-jaundiced neonates (*p* = 0.0004). • Male and female neonates with detectable AF and jaundice had lower birth weight than those without AF or jaundice (mean birth weight 55 g lower for males and 41 g for females, *p* < 0.05).
Kristensen	2000	56,720	Grain farming plus weather conditions as proxy for mycotoxin exposure	- Prenatal	Horticultural and agricultural census data	- Late-term miscarriage - Male urogenital defects a) Hypospadia b) Cryptorchidism	• Late-term miscarriage was more frequent in grain farmers with several seasonal fungal warnings as compared to those with one or no warnings (2 warnings: adj PR 1.80; 95% CI 1.1–2.9; ≥ 3 warnings: adj PR = 2.60; 95% CI 1.60–4.30). • Hypospadia was more frequent in grain farmers with ≥ 2 seasonal fungal warnings vs < 2 warnings (adj PR 1.80; 95% CI 0.90–3.40). • Cryptorchidism was more frequent in grain farmers with ≥ 2 seasonal fungal warnings vs < 2 warnings (adj PR = 1.2; 95% CI 0.60–2.40).
Moodley	2001	51	Fumonisin • FB_1_	- Prenatal	Maternal blood	- Pre-eclampsia - Eclampsia	• Fumonisin was detected in all maternal blood samples. • Mean blood fumonisin levels were higher in pre-eclamptic (0.45 ± 0.17 μg/ml; *p* = 0.009) and much higher in eclamptic pregnant women (2.85 ± 0.08 μg/ml; *p* < 0.001) compared to normotensive women (0.32 ± 0.08 μg/ml).
Abdulrazzaq	2002	201	Aflatoxins: • AFB_1_ • AFM_1_ • AFM_2_	- Postnatal	Cord blood	- Birth weight - Gestational age	• AF was detected in 71% of cord blood samples. • Prevalence and range of AFM_1_, AFM_2_ and AFB_1_ were 53%, 15%, and 35%, and concentrations ranged from 110 to 4060 pg/ml, 210 to 3700 pg/ml and 228 to 15,225 pg/ml, respectively. • Significant negative correlation (*r* = − 0.63; *p* < 0.001) between AF levels and birth weight. • Reported no correlation between AF levels and gestational age.
Abdulrazzaq	2004	166	Aflatoxin • AFM1	- Prenatal - Postnatal	- Maternal blood - Cord blood	- Birth weight - LBW - Neonatal jaundice	• AF was detected in 68% of maternal blood in concentrations from 30 to 8490 pg/ml and in 67% of cord blood in concentrations from 50 to 10,440 pg/ml. • Strong correlation between cord and maternal levels of AFM_1_ (*r* = 0.80; *p* = 0.001). • Strong negative associations between maternal AFM_1_ and birth weight (*r* = − 0.654, *p* < 0.001) and between cord AFM_1_ and birth weight (*r* = − 0.565; *p* = 0.001). • No evidence for a difference in incidence of jaundice between AFM-positive and -negative babies (15% vs. 13%, *p* = 0.69).
Nordby	2006	4912	Grain farming	- Prenatal	Horticultural and agricultural census data	Pre-eclampsia	• Pre-eclampsia was higher in animal farmers (aRR 1.14, 95% CI 1.07–1.22) and lower in grain farmers (aRR 0.93, 95% CI 0.86–1.01) compared to no animal and grain farmers respectively. • Pre-eclampsia was also lower in any year with two or more fungal forecasts appearing (RR 0.90, 95% CI 0.84–0.97) compared to years with no fungal forecasts.
Turner	2007	138	Aflatoxin: • AFB_1_	- Prenatal - Postnatal	- Maternal blood - Cord blood	- Birth weight - Weight gain - Height gain	• AF was detected in all maternal blood samples in concentrations from 4.8 to 260 pg/mg and in 48% of cord blood samples in concentrations from 5.0 to 89.6 pg/mg. • Weak positive correlation between cord and maternal levels of AF (*r* = 0.383, *p* < 0.001). • Infants from mothers with above median AF levels weighed 34 g less than infants with lower median maternal AF levels (*p* = 0.54). • Maternal AF was a strong predictor of infant weight and height gain (adj-*β* − 0.249, *p* = 0.012; adj-*β* − 0.207, *p* = 0.044).
Shuaib	2010	785	Aflatoxin: • AFB_1_	- Perinatal	- Maternal blood	- LBW - SGA - Preterm birth - Stillbirth	• AF was detectable in all maternal blood samples in concentrations ranging 0.44–268.73 pg/mg. • Non-significant positive association between highest quartile of maternal aflatoxin and SGA (adj OR 1.23, 95% CI 0.67–2.27), preterm birth (adj OR 1.30, 95% CI 0.75–2.27), and stillbirth (adj OR 1.35, 95% CI 0.52–3.50). • Significant positive association between “very high” maternal aflatoxin level and LBW (adj OR = 2.09; 95% CI 1.19–3.68).
Lauer	2018	220	Aflatoxin: • AFB_1_	- Prenatal	Maternal blood	- LBW- Small head size - Underweight- Stunting- Gestational age	• AF was detected in all maternal blood samples. • Elevated maternal AF levels were associated with LBW (adj-*β* − 0.07; 95% CI − 0.13 to − 0.003; *p* = 0.040) and lower head circumference *Z*-score (adj-*β* − 0.23; 95% CI − 0.43 to − 0.03; *p* = 0.023) at birth. • Non-significant association between maternal AF levels and infant length (adj-*β* − 0.10; 95% CI − 0.42 to 0.22; *p* = 0.53), WLZ (adj-*β* − 0.15; 95% CI − 0.40 to 0.11; *p* = 0.27), LAZ (adj-*β* − 0.07; 95% CI − 0.24 to 0.10; *p* = 0.41) and gestational age at birth (adj-*β* − 0.07; 95% CI − 0.41 to 0.26; *p* = 0.66).

*N* sample size, *AF* aflatoxin, *FB* fumonisin, *OTA* ochratoxin A, *LBW* low birth weight, *NTD* neural tube defect, *SGA* small-for-gestational age, *OR* odds ratio, *PR* prevalence ratio, *RR* relative risk, *sa:so* sphinganine-sphingosine ratio, *WLZ* weight-for-length *z*-scores, *LAZ* length-for-age *z*-scores, *Adj* adjusted, *ANOVA* analysis of variance, *CI* confidence interval

A modified Newcastle-Ottawa Scale for observational studies (Wells et al. n.d.) was used to assess the quality and risk of bias of the included studies. Two investigators (NNAK and DB) independently assessed each of the eligible studies against the following criteria: (a) representativeness of the sample, evaluated by the use of a random sampling method (b) valid and reliable exposure assessment, (c) valid and reliable ascertainment of outcomes, and (d) measurement of and adjustment for at least one key potential confounding variable in the multivariable models for observational studies. Each domain was awarded one point, giving a total of four achievable points—low scores indicate low quality and high risk of bias. Disagreements in the data extraction or quality assessment were resolved through further review and comprehensive discussion.

## Results

### Literature search

**Fig. 1:**
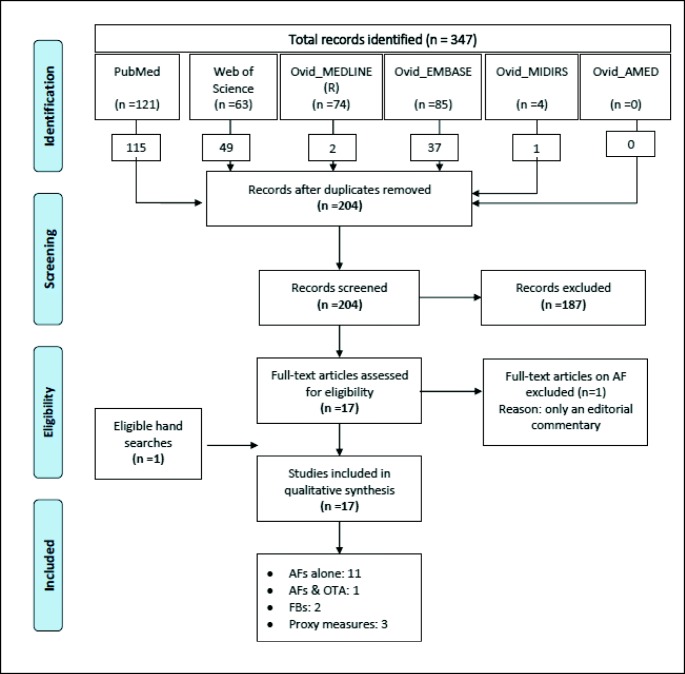
PRISMA flow diagram showing selection of the included studies. AFs aflatoxins, OTA ochratoxin A, FBs fumonisins

A total of 347 studies were identified through the systematic search. After excluding duplicates (*n* = 142), the remaining 204 studies were screened and 17 studies were then retained for full text review (Fig. 1). Of these, one study was excluded as it was only an editorial commentary. One additional eligible study was retrieved from hand searching. In total, 17 studies were included and appraised in this review. Eleven were on AFs alone, one was on AFs and OTA, two were on FBs, and three used grain farming and weather conditions as a proxy for mycotoxin exposure in general. None of the studies investigated the effect of DON or ZEN.

### Characteristics of the included studies

The 17 included studies were published between 1989 and 2018 (Table 1). The majority (10 studies) were conducted in the sub-Saharan African region (4 in Nigeria and 1 each in The Gambia, Sierra Leone, Ghana, Kenya, Uganda, and South Africa). Three further studies were from Europe, two from North America (United States and Mexico), and two were conducted in the United Arab Emirates. The studies employed cross-sectional (5/17), case-control (5/17), and cohort designs (8/17; of which 5 historical and 3 prospective). Only 5 studies reported the age of study participants and these included women in the reproductive age range of 15–45 years (Lauer et al. 2018; Missmer et al. 2006; Moodley et al. 2001; Nordby et al. 2006; Shuaib et al. 2010c). Out of a total quality score of 4, the median score for the included studies was 3, with an interquartile range (IQR) of 2 to 3.

Four studies used either information on agricultural/weather conditions or a validated aflatoxigenic food frequency questionnaire to assess maternal mycotoxin exposure, while most studies (13/17) quantitatively measured mycotoxin exposure; 11 of these used high-performance liquid chromatography (HPLC) often with fluorescence detection. Over half of the studies (9/17) assessed mycotoxins from maternal blood samples: four on the first antenatal visit (between 18 and 24 weeks of gestation) and five during the postnatal period. About half (5/9) of these studies also assessed mycotoxins from cord or infant blood samples. The remaining four studies that quantitatively measured mycotoxin exposure assessed mycotoxins either from cord blood or infant blood samples.

The median detection frequency of mycotoxins in maternal blood samples was 87%, with detection levels ranging between 17% and 100%. The median reported detection frequency of mycotoxins in infant blood samples was 48%, with reported levels ranging from 15% to 91%. The adverse pregnancy outcomes investigated were intrauterine growth restriction (10 studies), neonatal jaundice (4 studies), miscarriage, stillbirth or perinatal death (3 studies), pre-eclampsia (2 studies), spontaneous preterm birth (1 study), and birth defects (1 study).

### Mycotoxin exposures and pregnancy outcomes

Overall, ten studies were identified that evaluated the effects of maternal or fetal exposure to AFs on the occurrence of intrauterine growth restriction, which was mainly operationalized as lower mean birth weight (7 out of 10) and LBW (5 out of 10; Table 2).

Two studies examining mean birth weight found a negative correlation (*r* = − 0.63 and − 0.57) between AF exposure and birth weight (Abdulrazzaq et al. 2002, 2004), while three other studies reported lower mean birth weight (with the difference ranging from 34 g to 70 g) for infants with detectable AF levels (Abulu et al. 1998; Maxwell et al. 1994) or elevated maternal AF levels above median (Turner et al. 2007). A further study found a negative relationship only among female infants (with evidence for interaction) (De Vries et al. 1989). Only one study evaluated the joint effect of AFs and OTA on birth weight. Even though they reported no effect of the combined toxins on birth weight of boys, the mean birth weight of exposed girls was significantly lower (by 190 g) than that of unexposed girls (Jonsyn et al. 1995). All five studies that assessed effects on LBW found a positive association (OR_range_ = 1.07 to 2.29) between AF exposure and incidence of LBW babies (Abdulrazzaq et al. 2004; Carlos et al. 2014; Jonsyn et al. 1995; Lauer et al. 2018; Shuaib et al. 2010c).

Additionally, findings were suggestive of AF exposure triggering the development of neonatal jaundice. Two out of four studies found that the odds of AF exposure were more than two-fold (e.g. aOR = 2.68; 95% CI 1.18–6.10) in jaundiced neonates compared to non-jaundiced neonates (Abulu et al. 1998; Sodeinde et al. 1995). While the other two also found a higher incidence of neonatal jaundice among infants with AF exposure, these findings were also compatible with no difference between groups (Abdulrazzaq et al. 2004; Ahmed et al. 1995).

All three studies that evaluated the effect of a blood AF biomarker on gestational age or preterm birth found a higher likelihood of these outcomes among the exposed, but findings were also compatible with no difference (Abdulrazzaq et al. 2002; Lauer et al. 2018; Shuaib et al. 2010c).

No study on the effect of AF exposure on fetal loss or miscarriage was identified, but one study from Ghana found 35% higher odds of stillbirth at the highest level of maternal AF exposure, with a wide confidence interval, compatible with a more than three-fold increase and a halving of stillbirth (aOR = 1.35; 95% CI 0.52–3.50) (Shuaib et al. 2010c). The three large Norwegian studies that used grain farming and seasonal fungal warnings based on weather conditions as a proxy measure of mycotoxin exposure, found that such an exposure was associated with increased mid-pregnancy deliveries (gestational age 21–24 weeks; OR = 1.58; 95% CI 1.19–2.09) and late-term miscarriage (gestational age 16–27 weeks; OR = 1.31; 95% CI 1.11–1.55) but found no association with perinatal death (OR = 1.05; 95% CI 0.97–1.13) (Kristensen et al. 1997, 2000; Nordby et al. 2006). In contrast, a study from Mexico using aflatoxigenic food consumption as an exposure measure, found a positive association between maternal consumption of such foods with neonatal death (OR = 2.35; 95% CI 1.43–3.86) (Carlos et al. 2014).

One of the Norwegian studies using proxy measures for mycotoxin exposure also investigated hypospadia and cryptorchidism, urogenital birth defects among males, as outcomes. They found that these defects were more frequent (PR-hypospadia 1.80; 95% CI 0.90–3.40; PR-cryptorchidism 1.2; 95% CI 0.60–2.40) among children of grain farmers who were exposed to two or more fungal warnings in the season of conception compared to those exposed to one or no warnings, but findings were also consistent with no difference between groups (Kristensen et al. 2000).

Only two studies were identified on maternal FB exposure and adverse pregnancy outcomes (Missmer et al. 2006; Moodley et al. 2001). The study by Missmer et al. (2006) estimated maternal blood FB level using the blood sphinganine/ sphingosine (sa:so) ratio. This ratio was associated with an increased risk of neural tube defects (OR_range_ 1.5 to 4.5) such as spina bifida up to a threshold of sa:so ratio of 0.35, beyond which fetal death was more likely (Missmer et al. 2006). The study by Moodley et al. (2001) found that mean blood FB levels were lowest in normotensive pregnant women (0.32 ± 0.08 μg/ml), higher in pre-eclamptic women (0.45 ± 0.17 μg/ml), and substantially higher in eclamptic (2.85 ± 0.08 μg/ml) pregnant women (Moodley et al. 2001). In contrast, the study by Nordby et al. (2006) in Norway that used proxy measures for mycotoxin exposure (Nordby et al. 2006) found no association between grain farming and pre-eclampsia (aRR = 0.93; 95% CI 0.86–1.01) but did find an association between animal farming and pre-eclampsia (aRR = 1.14; 95% CI 1.07–1.22).

## Discussion

In this systematic review, the aim was to synthesize the available evidence from epidemiological studies on the effects of maternal or fetal exposure to mycotoxins on the occurrence of adverse pregnancy outcomes. Overall, this review found relatively few studies on the topic. The majority of studies focused on AF exposure, and results suggest detrimental effects of AFs on various pregnancy outcomes. Only two studies investigated the effects of *Fusarium* mycotoxins and found links to hypertensive emergencies of pregnancy and neural tube defects. Although exposure to multiple mycotoxins simultaneously is probably very common, only one study investigated the combined effect of AFs and OTA on intrauterine growth restriction.

The high mycotoxin levels detected in maternal and infant blood samples in the reviewed studies signify that mycotoxin exposure during pregnancy is widespread. Given this, the relatively limited number of studies identified in this review is concerning. The 17 studies were published within an almost 30-year span (between 1989 and 2018), with only three studies published in the last decade. Despite the wide geographic distribution of the included studies covering four of the six WHO world regions (Africa, Americas, Europe and Eastern-Mediterranean), it is noteworthy that no study was from the Southeast Asia region despite a large number of human mycotoxin biomonitoring studies in this region (Ali et al. 2016; Escriva et al. 2017). In Bangladesh, for example, human biomonitoring is common (Ali et al. 2016; Escriva et al. 2017) and studies show a high prevalence of mycotoxins among pregnant women (Ali et al. 2015, 2017), but the potential harmful effects on pregnancy outcomes have not been studied yet.

The methodological quality of the included studies is fairly good despite some notable limitations. For example, most studies (11/17) did not describe the population from which the study participants were selected or the criteria for selecting the analytic sample. Furthermore, because all the included studies were observational and many did not adjust for important factors such as maternal age and socioeconomic status, the possibility of residual confounding cannot be excluded. Nonetheless, all the included studies used an objective and reliable method for assessing adverse pregnancy outcomes and the majority (13 out of 17) also used standard quantitative methods for measuring mycotoxin exposure. The blood biomarkers used for AFs, for example, do not only provide an objective estimate of mycotoxin exposure but also provide an estimate of prior recent exposure (Turner et al. 2012). This is particularly important as it helps to establish temporality even in a cross-sectional or case-control design. Nevertheless, biomarker analyses are not a perfect way for assessing mycotoxin exposure, as blood levels depend on several factors, including characteristics of the specific toxin being measured, such as its bioavailability in tissues or biologic fluids and its detection window. As the detection window of most toxins is very narrow, only recent exposures can be accurately quantified, leading to the possibility of a misclassification error in relation to the etiologically relevant exposure, which may bias the results towards the null value. All the studies on AFs, however, used the blood biomarkers which reflect exposure over the previous 2 to 3 months (Turner et al. 2012). It is also unfortunate that hardly any of the included studies comprehensively described their exposure assessment, including information on volume of blood analyzed, limit of detection and limit of quantitation, and other specifics of the detection method used. This hampers reproducibility and detailed comparison of results. Additionally, most of the studies dichotomized the exposure based on the detection limit and did not study the effect of different levels of exposure, precluding the assessment of dose-response relationships.

### *Aspergillus* mycotoxins and adverse pregnancy outcomes

Three studies so far evaluated the effect of a blood AF biomarker on gestational age or preterm birth or on stillbirth (Abdulrazzaq et al. 2002; Lauer et al. 2018; Shuaib et al. 2010c) and found no evidence for an increased likelihood of these adverse pregnancy outcomes, albeit the trend went in this direction. However, considering the limited number of adverse events in these studies, they may have been underpowered. In terms of AF exposure and development of neonatal jaundice, there was some evidence of a detrimental effect despite limited sample sizes, with two out of four studies finding a clear increase and the other two trends in this direction.

There is comparatively good evidence for an adverse effect of maternal AF exposure on fetal growth, resulting in decreased mean birth weight and increased risk of low birth weight among exposed newborns. Two studies found this negative relationship only in female newborns. The others did not report sex-stratified results. It is noteworthy that two of the more recent studies that considered levels of AF exposure found a dose-response effect between AF exposure and fetal growth. However, almost half of the included studies did not sufficiently adjust for other known risk factors for intrauterine growth restriction such as maternal infections, maternal age, and socioeconomic status, which may be associated with AF exposure in pregnancy and thus lead to confounding. Nevertheless, the consistent evidence from animal studies of adverse effects of AFs on fetal growth, as well as on preterm deliveries and miscarriages (Ibeh and Saxena 1997; Ray et al. 1986; Wang et al. 2016), suggest that the observed associations in the studies reviewed are probably not just due to residual confounding.

Plausible biological mechanisms linking maternal exposure to *Aspergillus* mycotoxins to adverse pregnancy outcomes such as maternal anemia and intrauterine growth restriction have been previously described (Smith et al. 2017). Smith et al. (2017) suggest that mycotoxins affect pregnancy outcomes through three main pathways in both mother and fetus: (i) upregulation of pro-inflammatory cytokines and/or downregulation of anti-inflammatory cytokines, (ii) induction of enteropathy characterized by intestinal inflammation and impaired placental and fetal development, and (iii) toxic effects on fetal organs causing inflammation and impaired fetal development. Toxic effects of AFs on the newborn’s liver coupled with increased hemolysis of fetal hemoglobin may partly explain the apparent association between fetal AF exposure and neonatal jaundice. Teratogenic effects of OTA have also been documented in animals, with craniofacial abnormalities being the most frequently observed, alongside reduced birth weight (Malir et al. 2013). These harmful effects were shown to be potentiated by the presence of other mycotoxins or contaminants (Malir et al. 2013).

### *Fusarium* mycotoxins and adverse pregnancy outcomes

Various animal studies have linked adverse pregnancy outcomes such as neural tube defect and growth suppression to exposure to *Fusarium* mycotoxins (Gelineau-van Waes et al. 2012; Wolf and Horugel 1994). For example, there is strong evidence that FBs disrupt the biosynthesis of sphingolipids, vital structures in cell membranes, which interferes with folate receptors, affecting folate bioavailability and ultimately causing neural tube defects (Gelineau-van Waes et al. 2012; Marasas et al. 2004). In addition, there is evidence on the mechanism of toxicity of DON through its ability to inhibit ribosomal protein synthesis, as well as inducing systemic immune activation and alterations in the growth hormone system (Pestka 2010; Rotter et al. 1996).

This review found no human studies on maternal or fetal exposure to DON or ZEN and adverse pregnancy outcomes. This may partly be due to lack of valid biomarkers to accurately measure these exposures. Nonetheless, two studies were identified which evaluated maternal FB exposure and adverse pregnancy outcomes among maize-consuming populations in South Africa (Moodley et al. 2001) and North America (Missmer et al. 2006). These studies found an association with maternal exposure to FBs and increased occurrence of neural tube defect (Missmer et al. 2006) and pre-eclampsia and eclampsia (Moodley et al. 2001). The study by Moodley et al. (2001) assessed maternal exposure from the reported total number of corn tortillas consumed during the first trimester of pregnancy, which has potential for recall bias, but also used an objective measure of FB exposure obtained from maternal blood. Even though the findings of Missmer et al. are suggestive of a plausible association between maternal FB exposure and an increased occurrence of neural tube defect, temporality of recruitment and blood collection is arguably an important limitation. Animal studies suggest that following an exposure to a high dose of FBs, even sub-toxic doses will maintain an elevated sphinganine level (Enongene et al. 2002; Wang et al. 1999). Thus, postpartum levels might adequately reflect pregnancy levels if study participants are chronically exposed to FBs—a plausible situation in the study setting. Nevertheless, more rigorous prospective studies are needed to establish the effects of *Fusarium* mycotoxins on pregnancy outcomes.

### Proxy measures of mycotoxin exposure

While animal studies show a clear effect of mycotoxin exposure on pregnancy loss or miscarriage, there is limited evidence in humans. This may reflect a scarcity of methodologically rigorous pregnancy and birth surveillance systems to provide the needed information. Several large studies using grain farming and fungal warnings as proxy measures for mycotoxin exposure found associations with late-term miscarriage and mid-pregnancy deliveries. In contrast, studies measuring blood biomarkers of AFs were unable to provide evidence on any effects on preterm delivery or gestational age. These studies were mostly small, recruited women in late pregnancy, and lacked appropriate pregnancy and birth surveillance systems. Ideally, to ascertain true scientific evidence for the effect of mycotoxins on adverse pregnancy outcomes, a study will require a detailed record of events happening during the pregnancy period including all potential factors that my influence pregnancy outcomes. This is often impossible in most study settings. The availability of linked data, such as those provided by the Medical Birth Registry of Norway used in the proxy measures of mycotoxin exposure studies included, helps overcome this limitation and may partly explain the differences in findings. Furthermore, the small studies that used blood mycotoxin biomarkers also evaluated the effect of a single mycotoxin (AFs), rather than the effect of exposure to multiple mycotoxins as occurs in reality, which may be reflected by the proxy measures.

In Norway, where the proxy exposure studies were conducted, a recent study applying a semi-quantitative multimycotoxin analysis of grain grown in favorable fungal climatic conditions confirmed a high prevalence of multimycotoxin contamination (Uhlig et al. 2013). Nevertheless, the results from proxy measures are limited as they do not measure quantitatively the various mycotoxins present and also cannot examine how these mycotoxins may interact to influence adverse pregnancy outcomes. Furthermore, an aggregated area-level measure of possible mycotoxin exposure is an imperfect reflection of individual women’s exposures during pregnancy.

The differences in the prevailing types of mycotoxins in Norway and the African region may also partly explain the differences in findings from the proxy studies from Norway and the studies from Africa. For example, while the studies from Africa reflect a high prevalence of *Aspergillus* mycotoxins such as AFs, the study by Uhlig and colleagues showed a rather high prevalence and concentrations of *Fusarium* mycotoxins such as DON, FB, and ZEN in grain grown under exceptional climatic conditions in Norway (Uhlig et al. 2013).

### Strengths and limitations of this review

To our knowledge, this is the first systematic review synthesizing available epidemiologic evidence on mycotoxin exposure and pregnancy outcomes. Multiple relevant databases were systematically searched for relevant studies without the use of “filters” and language restrictions. Two researchers used predefined criteria to independently assess the eligibility of the identified studies and furthermore comprehensively assessed the quality of the included studies. Our systematic review considered a wide range of pregnancy outcomes, and the study was conducted in accordance with established guidelines for conducting systematic reviews.

Our study is however not without limitations. First, systematic reviews generally suffer from selective reporting of the original studies, given that null associations are often not published. Hence, selection bias cannot be ruled out. Second, this review unfortunately could not pool the results in a meta-analysis to estimate the effect of various mycotoxins on adverse pregnancy outcomes, because the included studies examined different mycotoxins and outcomes and their effect measures were differently reported, often without sufficient information for pooling.

In conclusion, fetal exposure to mycotoxins is widespread, especially in tropical low-income countries, indicating frequent mold contamination of maternal diets. In spite of this, there are relatively few epidemiological studies investigating the effects of mycotoxins on the occurrence of adverse pregnancy outcomes. Our systematic review, which synthesized the few available studies, found evidence for detrimental effects of (a) maternal exposure to AFs on intrauterine growth, potentially more so in girls, (b) fetal AF exposure on the development of neonatal jaundice, (c) maternal exposure to FBs on the development of hypertensive disease in pregnancy and neural tube defects, and (d) exposure to mycotoxins as measured through grain farming and weather conditions on preterm birth and late-term miscarriage.

More prospective studies, using pregnancy and birth surveillance systems or using linked databases, are still needed to firmly establish the effects of mycotoxin exposure on adverse pregnancy outcomes in humans. In particular, studies should take advantage of recent development in biomarker analysis, enabling relatively easy and objective measurement of multiple different mycotoxins that constitute real-life exposure.
